# A Parallel Process of Staff–Family Distress in Long-Term Care: A Challenge to Collaboration

**DOI:** 10.1177/23779608241306403

**Published:** 2024-12-15

**Authors:** Diandra Serrano, Tamara Sussman, Sharon Kaasalanien, Abigail Wickson-Griffiths, Genevieve Thompson, Paulette V. Hunter, Health B. MacIntosh, Kevin Brazil

**Affiliations:** 1 School of Social Work, 5620 McGill University, Montreal, QC, Canada; 2 School of Nursing, 62703 McMaster University, Hamilton, ON, Canada; 3 Faculty of Nursing, University of Regina, Regina, SK, Canada; 4 College of Nursing, Rady Faculty of Health Sciences, University of Manitoba, Winnipeg, MB, Canada; 5 St. Thomas More College: Psychology and Health Sciences, 7235 University of Saskatchewan, Saskatoon, SK, Canada; 6 130134 School of Nursing and Midwifery, Queen's University Belfast, Belfast, Northern Ireland

**Keywords:** Dementia < chronic illnesses, collaboration, long-term care, communication, end of life

## Abstract

**Introduction:**

Supporting persons living with advanced dementia in long-term care (LTC) homes requires strong collaborative partnerships between staff, family members, and residents. Yet, relational tensions—such as differing expectations around care decisions—can inhibit the implementation of collaborative partnerships at this critical point in the trajectory of care.

**Objective:**

This study aims to explore the emotional experiences of families and staff during shared decision-making processes for individuals with advanced dementia in LTC.

**Method:**

Guided by interpretative description, this qualitative study investigated the experiences of staff (*n* = 12) and families (*n *= 16) collaborating in two Canadian LTC homes. Data was collected through semistructured interviews lasting 45–60 min, which facilitated a detailed exploration of participants’ narratives. The interviews were audio-recorded, transcribed, and analyzed using reflexive thematic analysis facilitated by a combination of inductive and deductive approaches.

**Results:**

Our analysis revealed a complex parallel process of trauma and grief including accumulated distress, isolation, and feelings of devalue that worked together to create distance between staff and families at a time when connection was critical. Our findings further suggested that a lack of time and space for reflection and validation for staff and family, resulted in a cycle whereby staff and families engaged in a push and pull dynamic with each viewing the other as adversaries rather than allies.

**Conclusion:**

Our findings highlight the critical need for reflexive opportunities in LTC homes to overcome and attend to the emotional barriers that interfere with true collaboration between staff and families. We hope that the proposed cycle serves as a preliminary framework to support staff in navigating difficult conversations and emotions, and fosters reflexive care that enhances, rather than obstructs, connections.

## Introduction

As the prevalence and lifespan of dementia continues to rise globally, an increasing number of persons living at advanced stages of the condition will find themselves receiving end of life (EOL) care in long-term care (LTC) homes (Nichols et al., 2022; WHO, 2021). This context of care brings with it the need for strong family-staff collaboration around care planning and decision making (Hayward et al., 2021).

Existing studies have made valuable contributions toward understanding and informing what impedes and supports collaboration between families and staff who are implicated in caring for persons with advanced dementia in LTC (Davies et al., 2019; Hao & Ruggiano, 2020). However, studies have largely focused on practical and procedural aspects of collaboration and shared decision making with limited attention to how emotional experiences may impact these processes.

This qualitative study aims to fill this knowledge gap by examining the emotional experiences of staff and families during moments of attempted collaboration and shared decision making for persons living with advanced dementia in LTC.

## Review of the Literature

Dementia, a condition associated with myriad of cognitive diseases (e.g., Alzheimer), is globally recognized as a leading cause of death (Dumurgier & Sabia, 2021; Mathers et al., 2009). Persons in advanced stages of dementia often receive end-of-life (EOL) care in long-term care (LTC) homes because their care needs exceed the supports available in most Organization for Economic Co-Operation and Development (OECD) countries. Typical reasons for LTC entry amongst persons living with dementia include elevated caregiver burden, the need for daily support and monitoring, agitation and risk of harm to self or others if left unsupervised (Cable-Williams & Wilson, 2017; Canadian Institute for Health Information [CIHI], 2021).

Supporting persons living with advanced dementia brings about unique challenges such as attending to care needs and preferences amidst impaired verbal communication and judgement (Stokes et al., 2014). At these later stages, collaboration between LTC staff and families becomes increasingly critical (CIHI, 2021; Dupuis et al., 2016; Goodman et al., 2015). Yet, relational tensions between staff and families may be heightened at this demanding time, due to differing expectations around EOL care (Banerjee & Rewegan, 2016; Bennett et al., 2021; Jang, 2020) and structural pressures (Goodman et al., 2015).

To address the tensions related to care expectations, models of care interventions and frameworks referred to as family-centered care (Lopez et al., 2013), staff–family partnerships (Bauer & Nay, 2003; Jang, 2020), and shared decision making (Cranley et al., 2020; Egan et al., 2023) have been developed to help staff and families reflect on care circumstances and make joint decisions about goals of care. The overall spirit of these models is to encourage staff to view family members as a critical part of the care team, and help them to make care decisions that align with the perceived values and wishes of care recipients (Hoek et al., 2021). Shared decision-making models which offer the most explicit directions on how to facilitate inclusion, direct staff to present families with available care options, share their own knowledge, experience, and available evidence, and provide space and time to discuss care recipients’ values and preferences so that a joint care decision can be reached.

Although the literature documents the loss, burden and guilt that staff and families experience in the context of their respective roles, the extent to which these experiences may challenge motivations and efforts toward shared decision making on goals of care for the person living with advanced dementia have yet to be explored. This absence is particularly striking following the advent of COVID-19, where reports have repeatedly documented the high level of burnout, distress and even trauma experienced by staff in LTC homes, and the challenges faced by both families and staff when policies were put in place that undermined staff/family relations (Chu et al., 2023; Teixeira et al., 2022). For example, in many OECD countries family visitation was put to a halt and/or restricted in LTC for months, serving as a critical barrier to staff–family communication (Patton, 2022). Redressing this gap in the literature, the purpose of this study is to explore how, if at all, emotional experiences may be impacting collaboration and shared decision making between families and staff when supporting persons with advanced dementia in LTC.

## Methods and Methodology

### Study Design

We used an interpretive descriptive approach informed by the principles of reflexive thematic analysis to explore participants’ emotional experiences and examine how those reactions may have shaped staff/family caregiver interactions (Braun & Clarke, 2019; Braun et al., 2019; Byrne, 2022; Thorne, 2016). Interpretive description presumes the existence of multiple realities that are constructed through social interactions and influenced by context (Thorne, 2016). Researchers are hence expected to bring their expertise to the research process to support the development of rich interpretations that can be used to guide practice (Thorne, 2016). This approach aligns well with reflexive thematic analysis as both provide space for research to draw upon their own subjectivities to deepen the analytic process. Our collective expertise in social work, LTC, dementia care, relational connections and emotional responses allowed us to recognize, reflect on, and discuss the meanings we ascribed to participants’ accounts from our own subjective standpoints. This process resulted in a nuanced and contextually informed analysis.

Research questions:

In an effort to fill this gap in the literature and work toward informing models of collaboration that attend to the emotional experiences of both families and staff, this study sought to answer the following research questions:

1. What are the emotional experiences faced by families and staff during shared decision processes when supporting persons living with advanced dementia in LTC?
2. How do these experiences support or hinder staff–family collaboration on care decisions of residents living with advanced dementia in LTC?

### Recruitment and Sample

This study was conducted as a component of a larger transnational project called MySupport Family Carer Decision Support. The larger study aimed to develop, implement and evaluate an intervention to improve family members’ capacities to make EOL decisions when caring for persons living with advanced stages of dementia in LTC (Bavelaar et al., 2023; Brazil et al., 2024)**.** We focus here on data collected in the Canadian arm of the study capturing staff and families’ overall experiences with supporting the care of persons with advanced dementia in two LTC homes in Hamilton, Ontario and Montreal, Quebec.

### Inclusion/Exclusion Criteria

Families were included in the study if they were (a) 18+, (b) a primary caregiver, and (c) caring for someone living with advanced dementia in LTC. A lead staff person in each home initially approached families they judged to be eligible based on charting and clinical judgement to discuss their interest in participating in the project. Those who consented to learn more about the study were contacted by a research coordinator who provided further information and enrolled interested family members.

Staff were included in the study if they were (a) 18+, (b) working on a unit supporting persons with advanced dementia and (c) responsible for initiating or supervising care discussions with caregivers around EOL care. One lead staff person identified a mix of clinical/supervisory staff on each unit, informed them about the study via email and invited them to contact a member of the research team to learn more about participation.

### Data Collection

We conducted semistructured interviews with staff and families over an 18-month period between March 2021 and August 2022. This was a period when family visitation had actively recommenced following the restrictions imposed due to the COVID-19 pandemic. Four months prior to interviews, study sites, like other LTC homes across the country, were challenged to provide care amidst ministry-imposed rules on the use of personal protective gear, testing, and care priorities. LTC homes were also challenged to function with an untrained workforce called in to provide coverage for staff absenteeism and to develop systems for supporting family-resident communication when familial visits were forbidden (Hung et al., 2022; Wills et al., 2023). While usual care activities (i.e., regular care planning meetings, staffing, and visitation) had resumed during the data collection phase, the legacy of managing COVID related challenges was still looming.

A total of four research assistances (two per site) conducted all interviews. All research assistants were registered social workers or nurses who were hired by the research team during their graduate level studies. Interviews ranged from 45–60 min in duration. Staff interviews were conducted virtually at study inception and aimed to gain an understanding of staff's experiences supporting families of persons living with advanced dementia in care decisions. Staff interviews also sought to gather perceptions of the proposed intervention described elsewhere (Harding et al., 2022).

Family member interviews were conducted in a hybrid format either in-person in a private office in the LTC home or online. Family interviews were conducted following the completion of all staff interviews. While some nonverbal communication may have been lost with online interviews, offering both online and in-person options functioned as a crucial adaptation to ensure comfort and safety (Pocock et al., 2021) during a period of heightened stress postpandemic. These interviews explored family members’ overall experiences caring for persons living with end stage dementia in LTC. The interviews also sought to explore some of the main concerns family members hoped would be addressed in a structured meeting with staff. For this study we analyzed all staff and family accounts of their experiences (a) engaging in shared decision making processes to support persons living with advanced dementia in LTC and (b) collaborating with one another around EOL care planning. All participants provided their written consent prior to participating in an interview.

### Data Analysis

All interviews were audio-recorded, transcribed, and analyzed according to the principles of reflexive thematic analysis (Braun & Clarke, 2019; Braun et al., 2019; Byrne, 2022). Our reflexive thematic approach combined inductive and deductive methods. In the initial stages of analysis, we used an inductive approach so that we could immerse ourselves in the participants’ accounts.

To this end DS gained familiarity with the content of each interview by transcribing and then reading each transcript twice. Following this reading of the transcripts, DS and TS reflected on key ideas and meanings and jointly developed codes that seemed to represent the emotional challenges participants emphasized in the narratives. Some initial codes were developed such as loss and grief, exclusion, family reactivity, and staff avoidance.

DS and TS then reviewed all codes and associated excerpts together, and through discussion, reflection and active interpretation noted that many of the codes and excerpts capturing emotional and relational patterns aligned with two clinical concepts: parallel process and push and pull. We therefore turned to a more deductive approach as we felt some themes were best illuminated when interpreted through these theoretical ideas (Braun et al., 2022). For example, excerpts inductively coded as loss and grief, exclusion, family reactivity, and staff avoidance were reexamined deductively through the lens of parallel process, revealing more precise subthemes such as accumulated emotional suffering, feeling undervalued, pulling away by staff and pushing back by families.

As the interpretive process evolved DS and TS created reflected on and reviewed a draft report of the findings framed around the three core themes: parallel process, push-and-pull and viewing the other as adversary (the result of these processes). They also returned to the original transcripts to ensure interpretations captured key elements of participants’ narratives. It was through this process of writing, reflection, and reading that a missing contextual element to the analysis was inductively noted. Hence a fourth core theme was added to the findings: lack of time, an impasse, and opportunity.

In the final stages of the analytic process, DS disseminated the findings to other authors for further review, reflection, and refinement. This collective process of writing and review resulted in the slight renaming of core themes and subthemes, and the addition of a fifth theme labelled meaningful exceptions of positive dynamics.

An example of the data analysis process can be found in Table 1.

**Table 1.** An Example of the Analysis Process.

Citations (selected)	Initial codes (inductive analysis)	Emerging themes (emerging clinical concept)	Subtheme (deductively analyzing through the emerging clinical themes)
Staff member quote: “It's hard when you have your own loss and then seeing people who are not coping with their own loss (Ian).”	Loss and grief	Parallel process	Accumulated emotional suffering
Family member quote: “I don’t know what else to say to that other than it's created a great deal of stress and anxiety and guilt for me […] and it's awful (Stella).”	Stress, anxiety, and guilt		Accumulated emotional suffering
Staff member quote: “hearing that we’re having an [interdisciplinary team] meeting this week and the family wants to know why this medication was stopped. Well why wasn’t I there so I could have discussed that with the family? Now I have to call them again anyway (Christine).”	Exclusion		Feeling undervalued
Family member quote: “if they really valued me, they would be communicating with me (Harvey).”	Undervalued		Feeling undervalued
Family member quote: “There were other issues and nobody calls. […] You know, do they really want me to deal with it? […] I know how to deal with it. I can make it big; I can make phone calls. I can make a scene. I can call the head of the, you know, the building. I can call the ombudsman. […] You know, I can do all kinds of things, you know? Is this the way I really want to deal with it? Not really (Harvey).”	Family reactivity	Push and pull	Pushing back by families
Staff member quote: “like [staff] kind of just wonder what's the point, like they feel like they can't convince [families] anyways (Julie).”	Staff avoidance		Pulling away by staff

### Ethical Considerations

This research study was conducted in accordance with the standards of the Canada Tri-Council Policy Statement: Ethical Conduct for Research Involving Humans and was approved by the Office of Research Ethics Board Offices at the associated Integrated University Health and Social Services Centre in Montreal (Project 2022-2887) and McMaster University (Project 2019–5837).

## Results

### Study Participants

Sixteen out of 32 family members approached for this study participated in interviews. Their demographic details are presented in Table 2. The high proportion of adult daughters represented in our sample is similar to that reported in the literature for persons living with advanced dementia in LTC (Chappell et al., 2014).

**Table 2.** Family Member Participant Demographic and Caregiving History.

Characteristics	Sample
Site, n (%)	
Site 1	5 (31%)
Site 2	11 (69%)
Age (years)	
Mean (SD)	61 (8.47)
Range	51–70
Relationship to person with dementia, n (%)	
Spouse/partner	1 (6%)
Adult child	15 (94%)
Gender identity, n (%)	
Woman	12 (75%)
Man	4 (25%)

All 12 staff members approached for this study, participated in interviews (five from the first site and seven from the second). Their demographic information is detailed in Table 3. The variety of staff roles represented in our sample mirrors the variety of regulated nursing staff typical of care teams in LTC homes (McGregor, 2017; Squires et al., 2019).

**Table 3.** Staff Member Participant Demographic and Profession History.

Characteristics	Sample
Site, n (%)	
Site 1	5 (42%)
Site 2	7 (58%)
Age (years)	
Mean (SD)	49 (10.63)
Range	29–70
Professional role, n (%)	
Registered practical nurse (college degree)	3 (25%)
Registered nurse (bachelor's degree)	4 (33.33%)
Nurse practitioner (graduate degree)	1 (8.33%)
Clinical consultant	1 (8.33%)
Physician	1 (8.33%)
Care home manager	2 (16.68%)
Gender identity, n (%)	
Woman	10 (83%)
Man	2 (17%)
Years worked in LTC (years)	
Mean (SD)	12 (10.9)
Range	1month–12 years

*Note*. LTC = long term care.

### Themes

Our analysis of family and staff interviews revealed a complex parallel process of accumulated trauma, grief, and devaluation that worked together to create distance between staff and families at a time when connection was critical. Our findings further suggested that a lack of time and space for reflection and validation, resulted in a cycle whereby staff and families engaged in a push and pull dynamic which over time resulted in each viewing the other as adversaries rather than allies. These emotional and relational processes which appeared to inhibit rather than foster collaboration, are more fully depicted by the themes and associated excerpts presented below. Meaningful exceptions that hold promise for improving collaboration between families and staff at this critical time in care are also reported.

#### Theme 1: Emotional Experiences of Staff and Families: A Parallel Process

When describing overall experiences of providing care to persons living with advanced dementia in LTC homes, staff and families typically painted a portrait of accumulated suffering exacerbated in part by feeling unacknowledged and undervalued in their respective roles. Although the circumstances leading to these experiences differed, analysis of the data revealed striking parallels between the emotional demands and experiences of staff and those of families.

### Parallel Processes of Accumulated Emotional Suffering

Both staff and families entered the caring relationship with a history of accumulated distress that persisted from continued demands of care. For families, grieving the loss of their former relationship with their loved one with dementia, managing the residual guilt of needing to relocate their family member to LTC and being called upon to make EOL specific care decisions had a compounding emotional toll. One daughter who spoke of her experience directing staff to transition to comfort measures described her heightened emotions as follows:

It induced a lot of a guilt on my part because [my mother] was so angry. But I did everything they asked me to do and she is on, per her instructions, just the base […] I don’t know what else to say to that other than it's created a great deal of stress and anxiety and guilt for me […] and it's awful. It really is and it's not mom's fault and I feel bad for her but because I’m the only child, I’m the one who has to consistently make these decisions and you know it's impacting our life too. My husband and me […] we’re constantly on edge (Stella).

For staff, witnessing the dying process of residents, managing the accumulated suffering o families and living through their own experience of loss resulted in a level of suffering that was at times difficult to tolerate. As one nurse stated, “It's hard when you have your own loss and then seeing people who are not coping with their own loss (Ian).”

Another nurse described how COVID-19 amplified the preexisting distress felt by staff witnessing loss and grief; “We were just waiting when is this going to end when is it going to finish how is it going to be, everyday somebody else is dying (Maryam).” A second nurse, describing care experiences during the height of the COVID-19 pandemic stated, “it was war, I can only imagine that war is like that […] we did everything we could for them to be as comfortable as possible (Christine).”

Although many staff described the impacts of the pandemic on staff mental health, some also speculated that a clear understanding of its consequences could not yet be realized. One nurse described this as follows, “we don’t see the global picture still because as long as we’re in it I don’t think we’re going to talk about it really deeply (Cassandra).” The notion of not being able to process emotions while demands for care continue is noteworthy, as it is difficult to imagine when such a time will ever arrive.

Overall, both staff and families described an accumulation of ongoing suffering and distress that seemed to have no end in sight. Both also described a sense of doing everything humanly possible to offer support while feeling like their efforts were never quite enough.

### Parallel Processes of Feeling Undervalued

When asked about their involvement in EOL care decisions, staff and families both described experiences of being excluded from care decisions leading each to feel undervalued for their potential insights and contributions. For families, this process was fostered when they did not receive important information about their relative's care circumstances. For staff, it was heightened when they did not receive invitations to participate in care planning meetings that had been scheduled by other colleagues in the care team—resulting in feelings of exclusion.

Sharon, a daughter, described the irregularity in which staff kept her informed about her parent's care,

No, nobody keeps me informed about anything […] I mean keeping me informed would be like even a monthly report would be keeping me informed. I get a probably twice a year report. I don't know what they do there […] I think I'm extremely important to advocate for my mother's care. Do I think that the staff care that I'm there? No. as a matter of fact, I'm probably looked at as a, you know. Maybe… I don't know. I don't think staff care one way or the other.

Another son made the connection between the lack of communication by staff and the experience of being undervalued when he said, “if they really valued me, they would be communicating with me (Harvey).”

For staff, being excluded from potentially useful interactions with families was described as both frustrating and inefficient. One staff person who described the lack of information sharing within her team stated,

I feel like we would be able, in most situations, to answer most of [families] concerns […] there's a big portion that I feel that if we would have more information and more tools, we could support those families and not always waiting for the physician to have that conversation (Cassandra).

Another, describing their exclusion from a care planning meeting stated,

Hearing that we’re having an [interdisciplinary team] meeting this week and the family wants to know why this medication was stopped. Well why wasn’t I there so I could have discussed that with the family. Now I have to call them again anyway (Christine).

Contemplating the reasons why floor nurses might hesitate to discuss these topics with families, a nurse in a leadership role remarked,

Maybe sometimes [floor nurses] think we don’t consider their opinion, some of them, maybe that's why they don’t want to be involved […] Maybe that's why they are not confident, nobody has considered them, they don’t want to get involved (Maryam).

#### Theme 2: Staff–Family Dynamics: A Case of Push-and-Pull

While staff and families were experiencing emotions in parallel, these experiences appeared to distance rather than strengthen their connections to one another and their capacity to work collaboratively around care decisions. More specifically, the accumulated suffering and feelings of being undervalued described by staff and families alike seemed to contribute to staff's avoidance of families which in turn required families to push for involvement. These reactions set the stage for siloed approaches to care rather than collaborative decision making and partnership.

### Pulling Away by Staff

While in principle staff understood the valuable contributions of family members as the people who had the best interests of residents in mind, staff sometimes found it difficult to truly believe that engaging in such conversations would be fruitful in leading to an agreement in care. One staff member who speaks to this feeling of futility stated the following: “like [staff] kind of just wonder what's the point, like they feel like they can’t convince [families] anyways (Julie).” This sentiment suggests that the more heightened the distress of the family (those seemingly very distant from staff in terms of discussing care goals) the more likely staff were to avoid attempts at collaboration.

Staff's descriptions of avoidance were closely accompanied by a narrative that depicted families as having expectations that could never be fulfilled. One staff, Greg described how this sentiment could interfere with collaboration when he stated, “sometimes you have that feeling that no matter what you do, how much you try, you’re hitting a wall.”

Despite staff acknowledging that they did not have enough time or staff members on site, and that the space for collaborating with family members was suboptimal, their tendency to describe families’ needs as unrealistic expectations appeared to function as justification for staffs’ distancing.

### Pushing Back by Families

The combined distress of accumulated suffering and staff avoidance worked together to create a stance wherein some families felt they had to push back to remain involved in the care partnership.

This reaction is expressed by a son who had spent several days unsuccessfully trying to get a follow up on his mother's health:

There were other issues and nobody calls. […] You know, do they really want me to deal with it? […] I know how to deal with it. I can make it big; I can make phone calls. I can make a scene. I can call the head of the, you know, the building. I can call the ombudsman. […] You know, I can do all kinds of things, you know? Is this the way I really want to deal with it? Not really (Harvey).

A daughter, describing their readiness to push for information until satisfied stated, “I’m a dog with a bone […] If there's an issue or a challenge, I’m all over it until I get an answer to my satisfaction and if I don’t then it continues until I do (Stella).”

#### Theme 3: Viewing the Other as Adversary Rather than an Ally

Over time as both families and staff attempted to regulate the emotional demands they faced by pulling back (staff) or pushing forward (families) they began to internalize views of the other as burdensome and untrustworthy rather than as a trusting partner, making exchanges increasingly difficult.

One daughter who expressed feeling judged by staff when attempting to communicate her wishes for her mother's care recounted the following:

I had to really fight […] for certain things […] I would say, I don't want my mother force fed and then someone would say, Oh, so what, you don't want us to feed her. I just felt misunderstood sometimes and a bit judged (Nelly).

While another daughter described how lack of trust led her to engage in surveillance of staff:

I was really upset about a few things that were happening at night time and I went into my video at night time that I had videotaped […] and I sent it to the head nurse […] I am constantly checking, coming here or looking at the camera. and writing emails (Lorna).

Overall, many families expressed the necessity of engaging in fights with staff to have their opinions and preferences heard. This approach appeared to support a dynamic where staff who felt continuously questioned and monitored by families began to view families as self-centered, difficult and unrealistic. Eve's depiction of families as “very unrealistic [and] very egocentric” and John's description of families as “extraordinarily difficult” emphasize the positioning of family as a source of stress, rather than a partner in the care process.

#### Theme 4: Lack of Time, an Impasse, and Opportunity

The distress and interactions of most staff and family exchanges took place within a rigid environmental context that limited time to attend to unprocessed experiences, share information, and ultimately work together to make care decisions. Christine, a staff member, explained the pressures she and her colleagues are under when saying, “[staff are expected to] go, go, go … [and do not have] even 5 mins [to] sit down with [families].”

There was also a constraint on time that hindered staff's ability to address their own emotional processing. For instance, a manager expressed regret that their efforts to organize “healing circles (Greg)” for staff, providing an opportunity for collective expression of feelings, were not attended because staff perceived a shortage of time for such initiatives.

Without the time to recognize and process their own emotional needs, staff remained focused on their perceptions of families’ unrealistic expectations as propelling their need to pull away and set limits around their interactions. This resulted in families being seen as failing to understand the limits of the care system and positioned as overfocused on their relative's needs. The sentiments expressed by Eve depict this positioning of families:

Many times, the family members are very ego centric, what that means is that they only care about their loved ones […] you can’t really judge them for that, but we need to be fair to their loved ones in relation to the remaining population, given the current staffing ratio. They don’t always see that. It's all about my mom, my dad (Eve).

Quite possibly, functioning in an environment that deprived staff of the time and space they needed to recognize and process their own emotional challenges, supported both a growing sentiment of families as unreasonable and limited staff's capacity to acknowledge their own emotional activation.

Although the lack of time posed a contextual obstacle to enhancing understanding between staff and families, some moments of reflection on this external constraint seemed to create opportunity for understanding their collective suffering.

Several family members, while conveying their dissatisfaction with staff interactions, transitioned from viewing staff as uncaring to an understanding that the shortage of time was a significant external factor impacting staff's capacity to connect with them. For instance, a daughter, expressed,

I just don't think they care. I mean, as I said before that meeting, I think I think the nurses have to be more compassionate or more, the thing is they don't have time and I think it's not their fault (Marie).

Despite the prevailing sentiment among staff of families as demanding, a shift occurred when staff contemplated the essential role of time for families to manage their emotional experiences. One staff (Steven) noted, “family members that are very emotional are not able to sort of make decisions instrumentally because they are not able to cope.” While another staff explained, “family's perception as to what occurs is complicated by their emotional state when their relative is admitted […] it takes a lot of time […] you have to be very, very patient (John).”

Working within an environment where time was so scarce that staff felt squeezed to provide instrumental care seemed to provide staff with little time to meet family members’ emotional needs, let alone to recognize how their own unmet emotional needs might be contributing to and maintaining a push-and-pull dynamic. In contrast, moments of reflection on the external constraint of lack of time on their collaboration, presented opportunities to foster compassion and mobilize both staff and families toward a more understanding space.

#### Theme 5: Meaningful Exceptions of Positive Dynamics

Instances of positive experiences in shared decision making were particularly visible in two family members’ accounts. Several key gestures by staff, such as providing more supportive resources to families, developing familiarity with families over time, and making regular follow-up contact for reassurance, all seemed to enhance their feelings of being valued and make the emotional toll more tolerable for families.

For instance, a daughter described how she felt appreciated when staff provided a support group for family members. While another daughter described her ability to rely on a familiar staff member who had accompanied her along her 5-year journey with her loved one in the LTC home.

Being positively perceived by staff through gestures seemed to support these family members in reciprocally reflecting on staff member's needs. This was evident in Kyla's statement: “I’m a big advocate for every [personal support worker], every quarter to have a mandatory week's vacation and decompress. Mandatory.”

Feeling appreciated by staff also seemed to lead Lina into a reflective space where she could consider how best to approach collaboration with staff. She noted,

You know if you approach a question aggressively and in a negative way, you’re not going to get the support and the extra mile than if you’re calm and try to be understanding and listen to what they have to say. So, a lot of it depends on me. On how I approach challenges […] like they obviously don’t appreciate it so it's a two-way street.

Taking time to express small gestures of acknowledgement of family members by staff revealed a potential avenue for disrupting the push-and-pull cycle.

## Discussion

Families’ and staff's experiences interacting with one another to support residents living with advanced dementia in LTC, appeared fraught with emotional challenges that complicated their capacities to collaborate. More specifically, our findings revealed that both families and staff entered into their exchanges with a pile-up of emotional suffering and experiences of devaluation that propelled them into a dynamic of push-and-pull with each viewing the other as adversarial rather than ally. Our findings further illuminated that these stances were exacerbated by a systemic context that hindered their abilities to identify and manage their activated emotional states in ways that might have otherwise forged connections instead of distance. The mirroring of emotional experience, as presented in our theme “Emotional experiences of staff and families: A Parallel Process,” has been referred to in the psychoanalytic literature as a parallel process wherein a helper (in this case staff) unconsciously attributes their heightened emotions to characteristics and behaviors of the other (McNeill & Worthen, 1989; Morrissey & Tribe, 2001; So, 2013). When recognized this dynamic can generate heightened awareness, connection, and empathy between helper and helped. However, if unprocessed it can instead lead to a negative feedback loop wherein negative feelings and emotions are attributed to the ‘other’ and regulation is sought by the helper in ways that escalate rather than modulate the distress of the helped. Our findings suggested that the latter was a more common dynamic perpetuated in large part by a care context where staff and families felt devalued and excluded and where opportunities for emotional processing were rarely if ever available.

Feelings of exclusion reported by both staff and families, illustrated in our theme “Viewing the other as adversary,” appeared to reflect the consequences of a deeply embedded hierarchical structure within LTC contexts—a hierarchy that fails to equally value the contributions from all forms of caregivers within the collaborative care process and that consequently heightens relational tensions of domination and devaluation (Andersen, 2009).

Our findings further revealed that the absence of time and space to acknowledge these moments of deregulation led to the “pulling away by staff” in an attempt to find emotional reprieve and “pushing back by families” through pursuit. These findings draw similarities to current systemic models on emotional regulation that highlight the strategies that people engage in when trying to manage or find reprieve from intolerable feelings of distress (MacIntosh, 2019; Nolen-Hoeksema & Aldao, 2011). In this study, staff members engaged in strategies of labelling families as holding unrealistic expectations, solidifying their rationale for avoidance. With the constraints of time deeply ingrained in the fabric of the LTC, staff were deterred from reflecting on their own emotional experiences, impeding their abilities to respond to families with the flexibility, tolerance, and openness, that is critical for supporting mutual regulation (Butler & Randall, 2013; Butner et al., 2007). This led families to perceive staff as uncaring and even negligent, resulting in their need to pursue involvement through requests for information. When these efforts of pursuit failed some sought regulation through surveillance, seeking alternate medical opinions, and involving the media and legal services.

While the literature on staff–family relations in LTC extensively documents the stress experienced by both families and staff in LTC care contexts (Fetherstonhaugh et al., 2013; Lipnick et al., 2020; Samsi & Manthorpe, 2013), the dynamics revealed in our study present these experiences as relational, mutually reinforcing, and exacerbated over time by a care environment that sustained these distressing dynamics.

**Figure 1.**
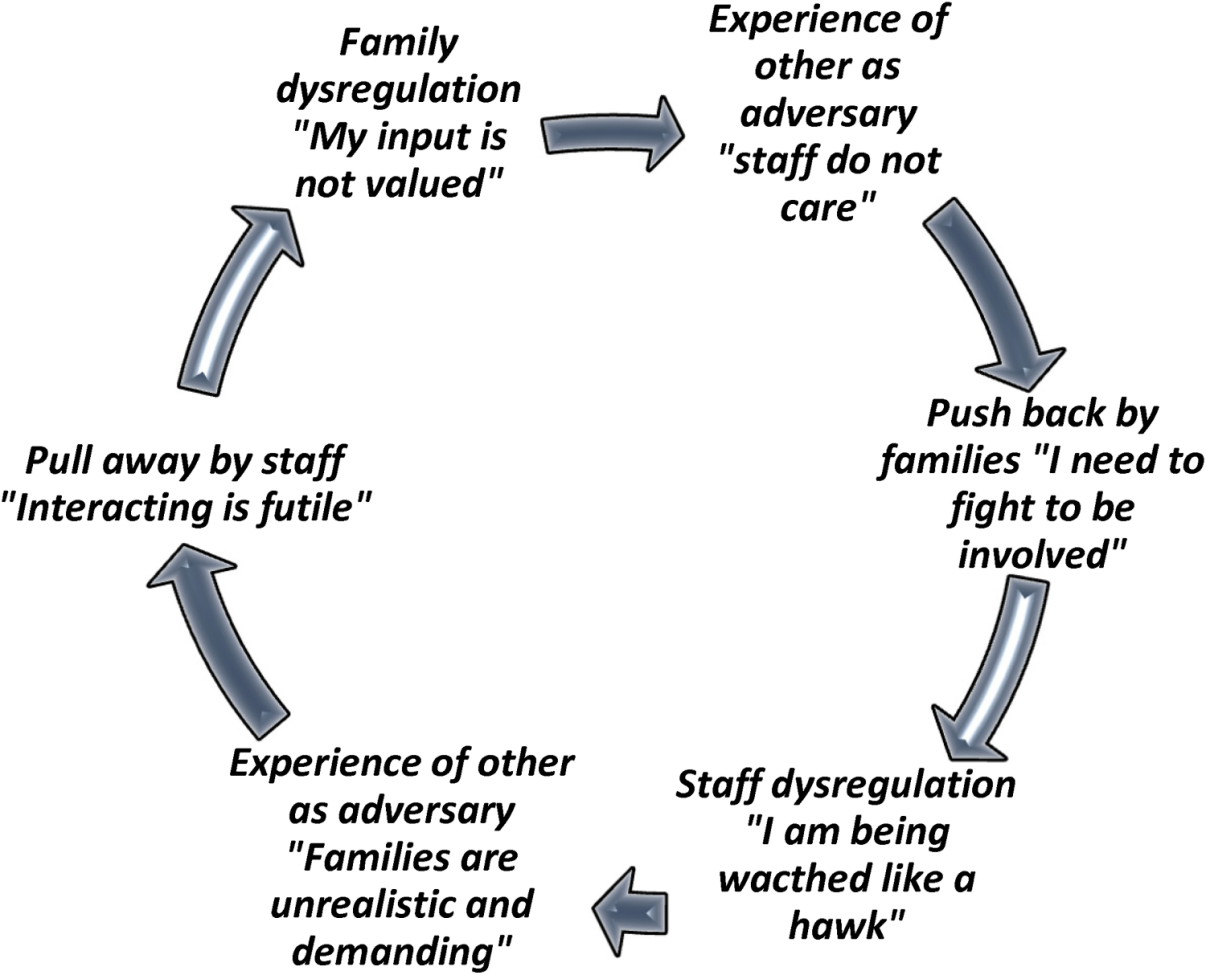
A Process of Push-and-Pull Supported by a Rigid System with Limited Time and Space for Reflection.

We have pictorially depicted how staff and family reactions to the emotional activation of the other (experienced in parallel), perpetuates emotional distress and results in an ongoing dynamic of push and pull in Figure 1. This figure also depicts that a rigid system of care that affords limited time and space for caring and reflective interactions can exacerbate and perpetuate these negative relational stances.

**Figure 2.**
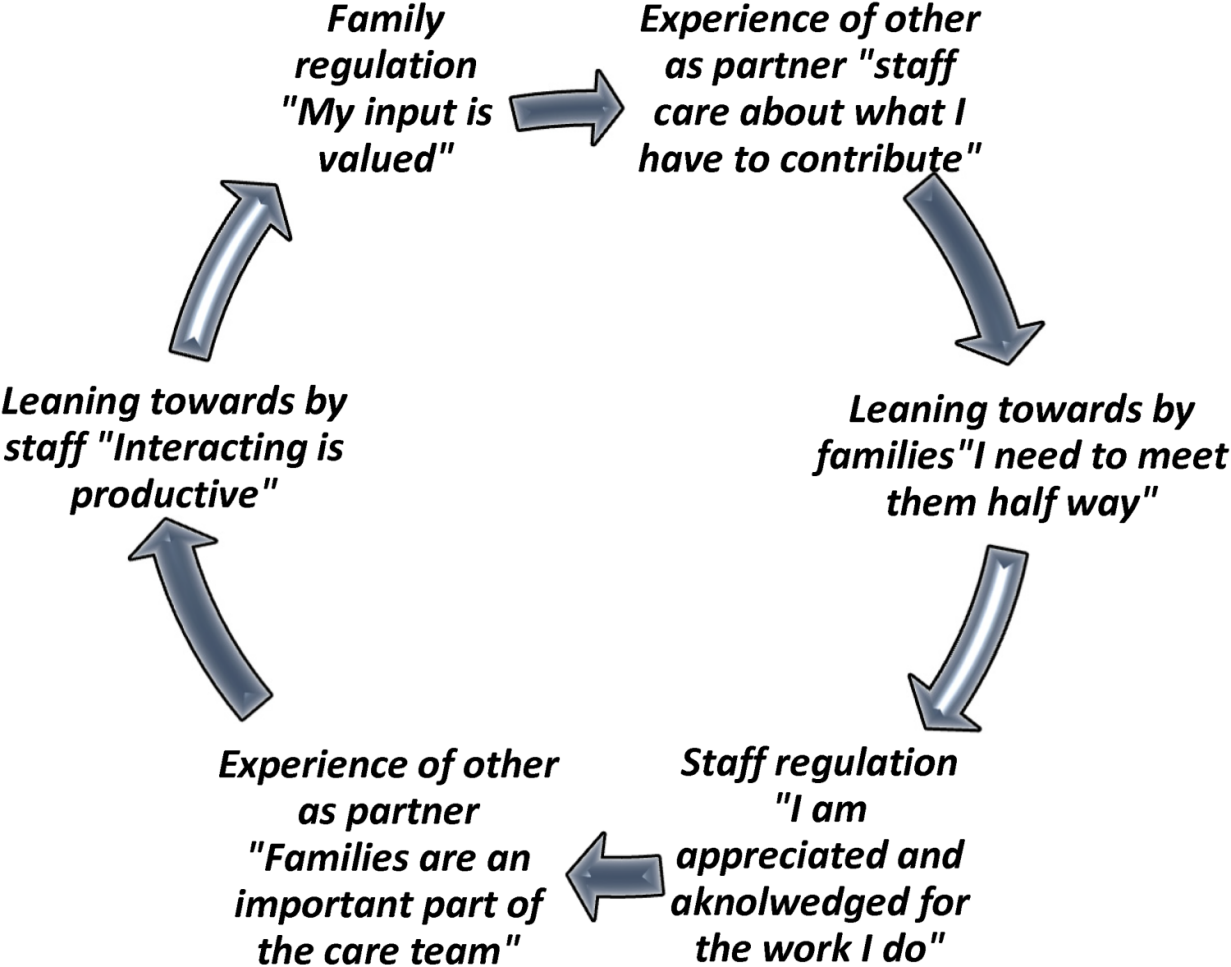
Disrupting the Push-and-Pull Cycle Through a Mutual Reflective Space.

Instances of contemplation were sometimes elicited during the interview process with participants which is captured the sub theme “lack of time, an impasse, and opportunity.” These moments were most apparent when staff and families were able to recognize the influence of inflexible time structures on one another's behaviors and experiences. Akin to the process of mentalizing or tuning into the thought processes of self and other to understand and change behavior, this process may have served as a first step toward disrupting the negative pattern of interaction between family and staff (Fonagy & Target, 2006; Macintosh, 2013). Unfortunately, these reflective mentalizing processes were rarely if ever supported by the broader system of care. Nonetheless, these meaningful exceptions provide us with an opportunity to envision the ways in which collaboration can be supported through spaces that attend to emotional distress and provide opportunity or mutual acknowledgement. Figure 2 draws from these positive interactions, offering a template for disrupting the push-and-pull cycle.

Our findings further suggest that the rules and regulation governing behaviors and interactions between staff and families within LTC may be limiting staff from engaging in a form of communicative action governed by mutual understandings between themselves and families (Hameras cited in Boye et al., 2021; Froggatt et al., 2011). For example, the value of efficiency within the social world of health care undoubtably contributes to staff managing emotional tensions by framing families as demanding, unrealistic and therefore interfering with their efforts to efficiently attend to their tasks. These sentiments appear to have grown rather than dissipated in the aftermath of COVID-19 supported by policies that positioned families as nonessential visitors rather than key partners in the care process (Patton, 2022). If staff and families were given opportunities to bridge their experiences (including collectively identifying the unrealistic demands placed on them by the current system of care) not only could they cultivate deeper alliances, but they may also engage in the form of collective resistance required to instigate change.

## Strengths and Limitations

This qualitative study offers important considerations for identifying and addressing the emotional experiences that may be impeding staff family collaboration and shared decision making in LTC for persons living with advanced dementia. However, the results of this work should be viewed in light of four limitations. First, the data informing this study was based on families’ and staff's reporting of their experiences supporting persons living with advance dementia in LTC as a part of a larger study. While the interviews revealed a series of dynamics that emerged between staff and families, observational data and more targeted questions on relational dynamics may have enhanced the rigor of our findings. Second, our study did not include the experiences of persons living with dementia who are critical participants in the lifeworld of LTC (Froggatt et al., 2011; Koster & Nies, 2022). Future observational studies examining the relational dynamics between staff and families could include the experiences of persons living with dementia (Soulières, 2019). Third, while our sample focused on regulated care staff who are typically assigned to hold EOL discussions with families, including perspectives from unregulated care staff—such as nursing assistants/aides—could provide additional insights. These unregulated staff who make up the largest portion of staffing in LTC (Andersen, 2009) might reveal other emotional challenges and demands not captured in our study. Finally, our study took place after the second wave of the COVID-19 pandemic (Ontario and Quebec, Canada). This meant staff who had been interviewed had observed high death tolls and some families had been subject to visitation restrictions. This context likely elevated the suffering experienced by families and staff in this study, heightened the strain on time, and exacerbated the need to coregulate via push-and-pull dynamic. However, as others have noted, the dynamics and challenges observed in LTC during the pandemic has served to expose cracks in a system that have been longstanding barriers (Banerjee & Rewegan, 2016).

## Practice Implications

Our findings reveal a series of implications for policy and practice that we believe may help to move practice forward. First, while interventions and educational initiatives continue to be developed and tested to improve staff's capacity to foster collaboration with families, our findings suggest that these more cognitively oriented approaches will do little to address the unconscious emotional states interfering with these forms of connections. While staff certainly suggested that knowing what to say may have helped them, taking the time to reflect on why they find it so difficult to engage with certain families would go a long way in making the unconscious conscious, thereby creating a space wherein staff can hold families within their distress. This means that training initiatives should focus on supporting reflexive practice, including providing staff with opportunities to identify their unacknowledged emotional reactions that activate a withdrawal response and work toward using these insights to foster closer connections with families (Vis et al., 2016). We hope that the cycles illustrated in Figures 1 and 2 will serve as clinical tools for staff to engage in reflective processing of their interactions with family members. These figures aim to enhance awareness of the emotions driving these dynamics, helping to transform unconscious patterns in more conscious, intentional engagement.

Second, a plethora of advocates have suggested that improving staff-to-resident ratios and work conditions must be a source of action if experiences of living and dying within LTC are to be improved (Armstrong et al., 2023; Glowinski et al., 2022). Our findings support and extend these calls by illuminating how lack of time and space, served to reinforce a circumstance wherein staff needed to develop adaptative strategies that distanced them from families. With more resources and supports available, the time staff so sorely needed to process their own reactions and remain present for families may be more feasible. Enhancing reflexivity requires allocating dedicated time for clinical reflection, such as through structured multidisciplinary reflection, role-plays, and regular debriefing opportunities. These practices can help staff recognize and manage their unconscious emotional responses, promoting more intentional and empathetic interactions with families.

Finally, the systemic focus on efficiency, which trickles into the cultural practices in LTC orients staff to prioritize doing over being (Froggatt et al., 2011). Within this environment it is no wonder that staff refuse to attend opportunities to debrief even if offered a chance to do so. Until more value is placed on attending to the emotional health and well-being of staff and families (Beck et al., 2012) we will continue to render staff and families susceptible to relating as adversaries rather than allies in times of high emotional stress and by so doing sustain a system of care which relies on this dynamic to retain the status quo.

## Conclusions

This study highlights the significant emotional complexities inherent in the collaboration and shared decision making between families and staff in LTC homes when supporting persons living with advanced dementia. The results reveal that fostering true collaboration between staff and families in LTC will necessitate more than the development of procedural /practical steps. We hope that our findings emphasize the critical need for staff and families to receive the time, space, organizational support, and reflexive training they require to work together rather than apart during this critical and complex time in the trajectory of care.

## Supplemental Material

sj-docx-1-son-10.1177_23779608241306403 - Supplemental material for A Parallel Process of Staff–Family Distress in Long-Term Care: A Challenge to Collaboration Supplemental material, sj-docx-1-son-10.1177_23779608241306403 for A Parallel Process of Staff–Family Distress in Long-Term Care: A Challenge to Collaboration by Diandra Serrano, Tamara Sussman, Sharon Kaasalanien, Abigail Wickson-Griffiths, Genevieve Thompson, Paulette V. Hunter, Health B. MacIntosh and Kevin Brazil in SAGE Open Nursing
